# The Misuse of Glasses

**Published:** 1902-09-06

**Authors:** 


### THE MISUSE OF GLASSES.

In an address delivered at Chicago Dr. F. C. Hotz,[1] draws attention to the mischief done by prescribing glasses for the relief of asthenopia in cases in which a more careful search might have revealed some pathological condition of the conjunctiva quite sufficient to account for the difficulty complained of. The point which he seems to make is this, that there are a large number of people going about in perfect comfort who, for all that, do not possess a degree of vision which is perfectly normal according to the tests of the ophthalmic surgeon. Their ametropia is perfectly capable of correction by appropriate glasses, but it does them no harm and at the most is less irksome than the wearing of spectacles. Should such a person, however, happen to be attacked with a conjunctivitis affecting the retrotarsal fold, where it is not easily seen, or with a slight degree of blepharitis, a certain difficulty of vision is at once produced, and if the physician, on discovering the refractional abnormality, should prescribe glasses —which he is very apt to do, either in consequence of not discovering the inflammatory complication or of wrongly regarding it as a consequence of the eye strain—he may not only fail to cure the complaint, but may do his patient considerable injury. As he says: "If such persons have faithfully used their glasses for a year or two without benefit, and if then they are relieved of their complaints in a week or two by the proper treatment of the blepharitis, and thereafter continue their work in perfect comfort, though they do not use glasses, we are forced to the conclusion that the glasses were unnecessary." The moral seems then to be the very old one, that we should not decide upon treatment without a full examination, especially of the retrotarsal fold and the margins of the lids, and that we should remember that moderate errors of refraction may be positively demonstrable, and yet not be the cause of the visual difficulty complained of.

[1] New York Medical News, Aug. 16.

